# Structural Colors Go Active

**DOI:** 10.1002/advs.202413027

**Published:** 2025-02-04

**Authors:** Xinting Li, Jiancun Zhao, Junyi Yang, Yihui Huo, Yiting Yu

**Affiliations:** ^1^ Ningbo Institute of Northwestern Polytechnical University College of Mechanical Engineering Northwestern Polytechnical University Xi'an 710072 China; ^2^ Key Laboratory of Micro/Nano Systems for Aerospace (Ministry of Education) Shaanxi Province Key Laboratory of Micro and Nano Electro‐Mechanical Systems Northwestern Polytechnical University Xi'an 710072 China; ^3^ Key Laboratory of Scale Manufacturing Technologies for High‐Performance MEMS Chips of Zhejiang Province Key Laboratory of Optical Microsystems and Application Technologies of Ningbo City Ningbo Institute of Northwestern Polytechnical University Ningbo 315103 China

**Keywords:** dynamic structural colors, fabry‐perot resonance, mie resonance, plasmonics color

## Abstract

Structural colors find wide applications for color printing, intelligent display, filtering imaging, etc., owing to their benefits, including high resolution, stable properties, and dynamic tunability. This review first illustrates the mechanisms of structural color generation, such as surface plasmon resonances, localized surface plasmon resonances, Fabry‐Perot resonances, Mie resonances, etc. It then proposes the recent technological strategies employed to realize dynamic structural colors. The integration of structural colors with functional materials like phase‐change, along with the development of color dynamic control mechanisms such as microfluidic chips, micro‐electro‐mechanical system drivers, and microheaters, represents key approaches for spectrum regulation. Furthermore, the review assesses the performance, advantages, and limitations of various technologies for dynamic structural colors. Finally, this review concluded with a section on the future challenges and prospects in large‐area fabrication, practical applications, and performance improvement. It explains the current typical applications, including smart windows, adaptive camouflage, sensors, etc., and explores the processing methods that can achieve large‐area, high‐fidelity preparation of structural colors, such as nanoimprint, deep ultraviolet lithography, immersion lithography, laser printing, etc. This field promises advancements in high‐density data storage, information encryption, and broader market applications.

## Graphical abstract

This review explores the principles and recent advances in dynamic structural colors, including SPR, LSPR, FP, and Mie resonances. It highlights key tuning strategies involving functional materials and external stimuli, evaluating their performance and limitations. With applications in displays, sensors, and anti‐counterfeiting, the review emphasizes future challenges in large‐area fabrication and practical implementations, envisioning widespread adoption.

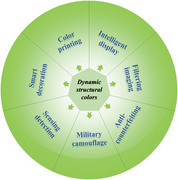

## 1 Introduction

Nature displays a vivid world with brilliant colors achieved through precise control of light‐matter interactions using optimized micro‐nano structures.^[^ ^1^, ^2^, ^3^, ^4^, ^5^, ^6^, ^7^, ^8^, ^9^, ^10^, ^11^, ^12^, ^13^, ^14^ ^]^ Colors in nature generally fall into two categories: structural color (physical color) and pigment color (chemical color). Pigments, such as those found in green leaves and flowers, achieve color by absorbing or reflecting light at specific. However, these chemical colorants exhibit chemical instability under high temperatures or intense ultraviolet light, and their limited resolution and durability constrain their use in advanced color displays and spectral imaging devices. Moreover, chemical colorants are expensive to produce and harm the environment when recycled. These disadvantages highlight the need for a new color generation platform that offers improved chemical stability, reduced toxicity, and higher resolution to meet the demands of innovative applications. The investigation of structural colors provides a viable solution to address these requirements.

The color generated by the interference, diffraction, or scattering of micro‐nano structures coupled with incident light is called structural color, which could be used to regulate the wavelength, amplitude, and polarization state of light accurately. Structural colors offer distinct advantages over chemical pigments, highlighted by the following features: 1) Enhanced color brilliance and a wide color gamut. By adjusting material properties and surface structures, structural colors enable precise selection of wavelengths, achieving customized colors as needed. 2) Higher resolution and integration.^[^ ^11^, ^12^, ^15^, ^16^, ^17^, ^18^, ^19^, ^20^, ^21^, ^22^, ^23^, ^24^, ^25^ ^]^ Currently, conventional pigment printing resolutions are typically ≈1200 dpi, while resolutions of structural color printing can reach 100 000 dpi.^[^ ^26^ ^]^ Additionally, the Integrated Circuit (IC) technique, which has strong compatibility with photoelectric devices, can be integrated with the structural colors generated using silicon (Si), aluminum (Al), and other materials. 3) Stable physical and chemical properties. Due to their inherent physical characteristics, structural colors exhibit resilience against radiation and corrosion, even in harsh environmental conditions. 4) Green, clean, and pollution‐free. Compared to chemical colors, such as pigments, structural colors are inherently more environmentally friendly, as they do not rely on chemical dyes or pigments that can contribute to pollution. While the fabrication of structural colors may still involve energy consumption and certain chemicals, the resulting structures themselves are free from the environmental concerns associated with traditional chemical coloring methods. 5) Dynamic tunability. Unlike pigment colors, structural colors can be controlled by external stimuli such as heat, pressure, light, or electricity. In summary, structural colors represent a promising alternative to chemical pigments, offering improved performance across various parameters crucial for advanced applications.

**Figure 1:**
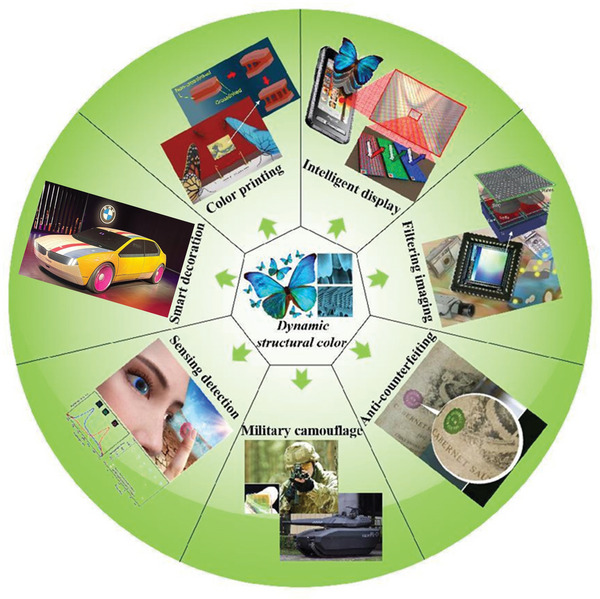
The wide application fields of dynamic structural colors.

The dynamic tunable property of structural colors gives them great potential in wide application fields,^[^ ^27^, ^28^, ^29^, ^30^, ^31^, ^32^, ^33^, ^34^, ^35^, ^36^, ^37^, ^38^, ^39^ ^]^ such as color printing, intelligent high‐resolution display, filtering imaging, anti‐counterfeiting, military camouflage, sensing detection, smart decoration, and more, as shown in **Figure** **1**. Samsung has innovated by employing structural color in organic electroluminescence displays (OLED), enhancing resolution to 10000 dpi and surpassing traditional pixel density limitations.^[^ ^36^ ^]^ The use of structural color printing in glassy polymer films, as proposed by Masateru M. Ito et al., involves controlling microfibril and cavity formation within the film by use of standing‐wave optics.^[^ ^35^ ^]^ The structured stress micro‐fibrillation process can be turned into a large‐scale, inkless color printing method by utilizing regular lithographic and masking equipment. This creates images in a range of flexible and transparent formats with resolutions up to 14 000 dpi. Yu Horie et al. proposed visible wavelength color filters using dielectric subwavelength gratings for backside‐illuminated complementary‐metal‐oxide‐semiconductor (CMOS) imaging sensor technologies.^[^ ^37^ ^]^ To realize the instrument‐free detection of pathologically relevant signals of eye diseases, Yunlong Wang et al. established a structurally colored contact lens sensor with a tunable color, which, by changing colors, can instantly indicate changes in moisture and pressure—two factors that are crucial for diagnosing xerophthalmia and glaucoma, respectively.^[^ ^38^ ^]^ Jehyoung Koo et al. have created a highly flexible dynamic multicolor electrochromic skin (DMECS) inspired by cuttlefish skin patterns, paving the way for adaptive camouflage technologies.^[^ ^39^ ^]^ Furthermore, structural colors find applications in medical diagnostics, food detection, and beyond. To meet practical application needs, further research is necessary on the performance and regulatory mechanisms of structural colors.

As nanofabrication and characterization technologies advance quickly, the dynamic structural colors and related nanodevices have already been investigated intensively in recent years.^[^ ^40^, ^41^, ^42^, ^43^, ^44^, ^45^ ^]^ A series of dynamic structural colors have been prepared by studying the composition materials and micro‐nano structures of natural structural colors. This review summarizes recent advancements in dynamic structural color technologies. First, we introduce the design principles of structural colors and highlight key factors, such as geometric parameters and material properties, which can be manipulated to control filtering properties. Second, we summarize and analyze recent research on changing the above factors by applying external stimuli such as optical, electric, magnetic, thermal, and many other physical, chemical, and biological factors, or by adding new functional materials such as 2D materials or phase‐change materials to get the dynamically tunable structural colors. Lastly, we discuss future prospects for practical applications, fabrication, and enhancing the performance of dynamic structural colors. Overall, these advancements primarily leverage plasmonic and photonic structures, which have gained popularity over the past two decades.

## 2 Principles for Structural Colors

Nanostructures and their combinations can manipulate visible light through scattering and absorption, either enhancing or suppressing specific bands, thereby creating distinct colors. While various methods exist for achieving these spectral modulations, they all rely on the introduction of a series of resonances that induce significant phase shifts. There are mainly the following types: surface plasmon resonances (SPR),^[^ ^12^, ^26^, ^46^, ^47^, ^48^, ^49^ ^]^ localized surface plasmon resonances (LSPR),^[^ ^50^, ^51^, ^52^, ^53^ ^]^ Fabry‐Perot (FP) resonances,^[^ ^54^, ^55^, ^56^, ^57^, ^58^, ^59^, ^60^, ^61^, ^62^, ^63^, ^64^, ^65^, ^66^, ^67^, ^68^ ^]^ Mie resonances,^[^ ^69^, ^70^, ^71^, ^72^ ^]^ etc.

In order to more clearly and intuitively understand the key features and differences of color generation based on the above resonance modes, **Table** **1** lists and compares them.

**Table 1:** Comparison of resonance modes to achieve structural colors.

Resonances	Structures	Key factors	Advantages	Limitations	Applications	Refs.
SPR	Metal film‐dielectric	1) Geometric parameters (metal thickness) 2) Material properties 3) Incident light conditions 4) External environment (temperature, pH, etc.)	1) High sensitivity 2) Wide spectrum control 3) Simple to integrate	1) Angle‐ sensitivity 2) Fabrication complexity 3) Low stability	1) Sensors 2) Decoration 3) Anti‐counterfeiting	[12, 26, 46, 47, 48, 49]
LSPR	1) Nanometal particles 2) Nanohole arrays	1) Geometric parameters (size, shape, and gap) 2) Material properties 3) Incident light conditions 4) External environment (temperature, pH, etc.)	1) Extremely small size 2) High saturation 3) High resolution 4) Dynamic control 5) Wide spectral range	1) High metal loss 2) Low stability 3) Angle sensitivity 4) Fabrication complexity	1) Nano‐optical devices 2) High‐resolution displays	[50, 51, 52, 53]
FP	Metal‐ dielectric‐ Metal	1) Material properties 2) Thickness	1) Precise control 2) Large angle‐insensitive 3) Flexible design 4) Fabrication simply	1) High reflectivity 2) Material restrictions	Optical filters	[54, 55, 56, 57, 58, 59, 60, 61, 62, 63, 64, 65, 66, 67, 68]
Mie	1) All‐dielectric nanoparticles 2) All‐dielectric array	1) Geometric parameters 2) Material properties 3) Surrounding medium 4) Incident light conditions	1) Low optical loss 2) Strong durability 3) Wide color gamut 4) Large‐area fabrication	1) Fabrication complexity 2) Angle sensitivity 3) Material restrictions 4) Environmental sensitivity	1) Durable optics 2) Environmental sensors	[69, 70, 71, 72]

### 2.1 SPR and LSPR

Plasmonic structural colors can be generated from LSPR, propagating surface plasmon, plasmon hybridization, and the coupling between the surface plasmon and other optical modes.

**Figure 2:**
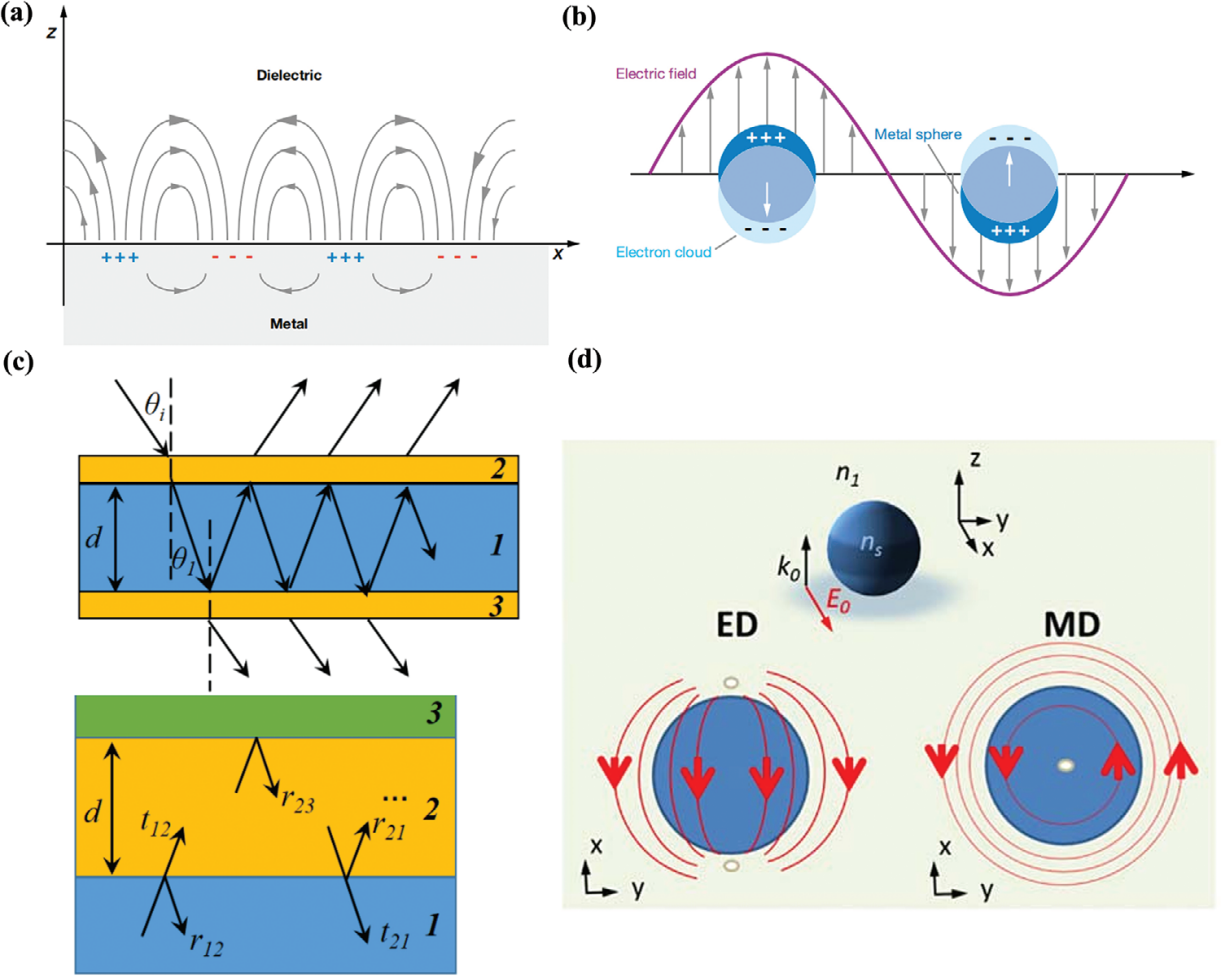
Schematic diagram of color generation principle. (a) SPR. (b) LSPR.^[^ ^73^ ^]^ Copyright 2023, Licensee MDPI, Basel, Switzerland. (c) FP resonance. (d) ED and MD resonances.^[^ ^82^ ^]^ Copyright 2017, OSA.

The distinction between propagating and confined surface plasmons is seen in **Figure** **2**a,b.^[^ ^73^ ^]^ As shown in Figure 2a, surface plasmons (SPs) are collective free‐electron oscillations at the metal‐dielectric interfaces.^[^ ^73^ ^]^ At the resonance wavelength, they show an enhanced near‐field amplitude of the electric field, which is highly confined and decays exponentially away from the interface. Plasmons travel along the metal‐dielectric contact in both x and y directions for distances ranging from tens to hundreds of microns. They then decay evanescently in the z direction, with a 1/e decay length on the order of 100 nm.^[^ ^73^ ^]^ The incident angle, polarization, and environmental index are some of the variables that can be used to adjust the SPR. Different materials and boundary conditions allow for the excitation of different plasmon modes that have various polarization, phase, and resonant frequencies.^[^ ^74^ ^]^ New interest in SPs has been generated by the demonstration of improved transmission through periodic arrays of subwavelength holes in optically thick metallic films. Both theoretically and experimentally, hole arrays have been extensively studied, particularly in their selective extraordinary transmission for color filtering purposes. The wavelength of the transmission peak *λ*_max_ depends on the periodicity and constituent of hole arrays, as well as the structural symmetry.

The periodic nanohole array that harvests the incident light through the optically metallic film enhances the evanescent wave electric field, thereby increasing the incident light transmittance and generating resonance on the surface via the hole. The periodic arrays in subwavelength‐thick metallic films determine the resonant modes. As for a square array and a hexagonal array, the relationships between the peak wavelengths *λ*_max_ and the geometric parameters of the array can be given by Equations (1) and (2), respectively. For a square array of period P, *λ*_max_ of transmittance spectra at normal incidence can be identified approximately from the dispersion relationship (Equation (1)), and is given by:

(1) $$\lambda_{\max(i,\;j)}=\frac{P}{\sqrt{i^{2}+j^{2}}}\sqrt{\frac{\varepsilon_{m}\varepsilon_{d}}{\varepsilon_{m}+\varepsilon_{d}}}$$

and for the hexagonal array:

(2) $$\lambda_{\max}=\frac{P}{\sqrt{\frac{4}{3}}\left(i^{2}+ij+j^{2}\right)}\sqrt{\frac{\varepsilon_{m}\varepsilon_{d}}{\varepsilon_{m}+\varepsilon_{d}}}$$

where P refers to the array period, *i* and *j* refer to the scattering orders from the array. ε_m_ and ε_d_ represent the index of metal and dielectric, respectively.

For the case of localized surface plasmons, as shown in Figure 2b, light interacts with nanoparticles much smaller than the incident wavelength. As a result, a plasmon with a frequency known as the LSPR oscillates locally around the nanoparticle. LSPR, similar to SPR, is sensitive to changes in the local dielectric environment. Typically, the LSPR wavelength‐shift measurement is used by researchers to detect changes in the local environment. Metallic nanocrystals can be seen as subwavelength optical antennas. Their material, size, geometry, surroundings, and interparticle distance all have an impact on how sensitively they support LSPR. Thus, by modifying the nanoantenna's characteristics, one can achieve the required transmission or reflection spectra.

In addition to the nanoantenna arrays, metallic grating nanostructures and nanohole arrays on metallic films are also good candidates for plasmonic color generation because of their selective color filtering or reflection, which usually comes from the hybridization of LSPR and propagating surface plasmons.^[^ ^75^, ^76^ ^]^ In short, the dynamic structural colors can be regulated and controlled by changing the external environment, such as the polarization state, angle of incident light, and surrounding environment, particle morphology or size, and the distance between particles, as well as material properties.

### 2.2 FP Resonance

Thin‐film interference is perhaps the simplest source of structural colors, which arises when an incident light wave is reflected at each boundary of a thin film, resulting in the interference of two reflected waves to form a new wave.^[^ ^77^, ^78^ ^]^ The FP optical cavity, typically composed of a wavelength‐scale‐thick transparent or lossless medium sandwiched between two highly reflective surfaces, has played a crucial role in diverse fields, including lasers, spectroscopy, modulators, and tunable filters. Depending on the thickness and optical characteristics of the cavity medium, a particular wavelength (i.e., resonance) can create standing waves due to multiple reflections of the light within the cavity. As shown in Figure 2c, the resonance occurs when the sum of the propagation phase changes and the two reflection phase changes is equal to 2 mπ. The FP resonances will be formed in the optical spectrum and produce the reflective colors.^[^ ^79^ ^]^

(3) $$\varphi_{12}+\varphi_{23}+\varphi_{pro}=2m\pi$$

(4) $$\varphi_{\mathrm{pro}}=\frac{2\pi n_{1}}{\lambda }2\mathrm{dcos}\left(\vartheta_{1}\right)$$

where φ_12_ is the phase shift occurring due to the absorption in the top metallic film; φ_23_ is the phase shift occurring upon the reflection at the bottom metallic reflective film; φ_pro_ is the total of the propagation phase shift in the transparent dielectric between the metallic films; *n*_1_ is the index of layer 1.

The refractive index of the medium layer can be altered through heating, or the cavity length can be adjusted via thermal expansion. Changes in the surrounding environment can also affect the refractive index of the medium layer or the cavity length. Beyond single‐factor adjustments, various combined regulatory mechanisms could enhance color‐changing performance significantly.

### 2.3 Mie Resonance

Mie resonances refer to a series of optical resonance phenomena caused by the interaction between electromagnetic waves and subwavelength‐scale dielectric particles.^[^ ^20^, ^80^, ^81^ ^]^ Its notable feature is the simultaneous excitation of multi‐level resonance modes, including electric dipoles (ED) and magnetic dipoles (MD), as shown in Figure 2d.^[^ ^82^ ^]^ The dispersion of the light field within the medium is responsible for these patterns. Rich structural colors can be produced by selectively enhancing the scattering or absorption of specific wavelengths of light through precise control of the geometric parameters and material properties of the particles. ED resonance occurs when the electric field induces a distribution of dipole charges within the particles during their interaction with incident light. This resonance is characterized by strong scattering and polarization features, as well as a symmetric electric field distribution around the particles along the path of light propagation. It is primarily influenced by the dielectric constant and particle size. ED resonance is often more pronounced in smaller particles. MD resonance arises when a ring‐shaped current distribution is induced inside the particles by the incident light, creating a magnetic field‐like effect that manifests as MD resonance. The MD resonance depends on the high refractive index of the particle material and the appropriate size ratio. When the particle size approaches a specific fraction of the wavelength of the incident light, MD resonance is significantly enhanced. This mode is a unique characteristic of Mie resonances, fundamentally different from the SPR of traditional metal nanoparticles. In addition to the basic ED and MD modes, Mie resonances can also excite higher‐order resonant modes, such as electric and magnetic quadrupoles. These modes are associated with more complex multipole moments and intricate light field distributions. The excitation of higher‐order modes is typically linked to an increase in particle size, offering more precise control over the directionality of light scattering.

Mie resonances have unique advantages in the generation of structural colors. Compared with structures based on plasma resonances, dielectric materials exhibit low absorption losses in Mie resonances, which makes them have higher optical efficiency and stability. In addition, the Mie resonance mode exhibits unique characteristics in MD resonance, which can effectively enhance the interaction between light and materials, thereby achieving color modulation in a wide spectral range. Due to its strong designability and efficient light control ability, structural colors based on Mie resonances have shown broad application prospects in the fields of display technology, anti‐counterfeiting technology, and sensors. For example, by adjusting the geometric size and periodic distribution of the nanostructure, an optical display device with high resolution, high saturation, and low angle sensitivity can be developed. This flexible regulation capability makes Mie resonances an important research direction in the field of structural colors.

## 3 Dynamic Structural Colors

Accordingly, we analyzed several resonance modes (SPR, LSPR, FP, and Mie resonances, etc.) for producing structural colors and learned about the key factors that determine the color performance of structural colors. Dynamic structural colors can be achieved by changing the key factors through various tuning methods.^[^ ^83^, ^84^, ^85^, ^86^, ^87^, ^88^, ^89^, ^90^, ^91^, ^92^, ^93^, ^94^, ^95^ ^]^ First, we will classify them based on the resonance methods of achieving color and then further refine the key factors of the device that are regulated to achieve dynamic structural colors, such as material properties, geometric parameters, incident light conditions, external environments, etc.

### 3.1 SPR‐/LSPR‐Based Structural Colors

Based on the SPR/LSPR resonance effect, the geometric parameters of nanostructures (size, shape, and gap) play a critical role in determining the resulting resonance frequencies and, consequently, the observed colors. By introducing various tuning methods—such as mechanical deformation, electric fields, etc.—it is possible to dynamically alter these geometric parameters. This modulation of size, shape, or gap in turn shifts the resonance frequencies, enabling real‐time and reversible tuning of structural colors.

**Figure 3:**
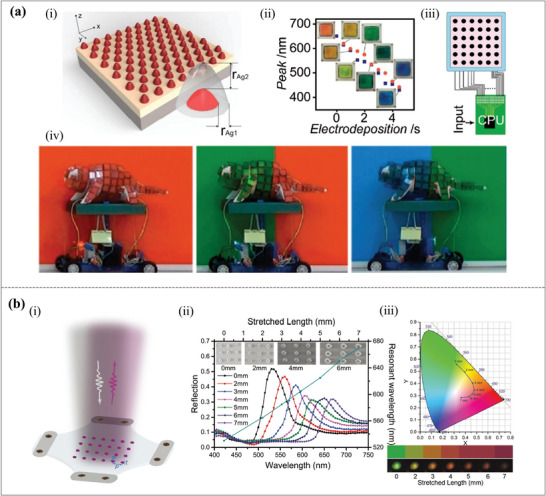
Dynamic structural colors are caused by changing geometric parameters of plasmonic structures. (a) Mechanical chameleons through dynamic real‐time plasmonic tuning with reversible electrodeposition. (i) Schematic of the simulation and related parameters. (ii) The blue square represents the electrode placements, and the orange dot represents the electro‐stripping times that determine the reflection peak wavelength. Images of the gadgets that correspond to the chosen points are included as insets. (iii) Operational schematic of the “fast display”. (iv) Screenshot of the plasmonic chameleon demonstration movie.^[^ ^96^ ^]^ Copyright 2016, American Chemical Society. (b) Actively tunable structural color rendering with tensile substrate. (i) Schematic of the dynamic tuning structures based on PDMS substrate. (ii) Measured reflective spectra with different stretched lengths, along with the corresponding SEM images in the insets. (iii) The calculated CIE chromaticity diagram of the reflection spectra in (ii).^[^ ^97^ ^]^ Copyright 2019, Wiley‐VCH Verlag GmbH & Co. KGaA, Weinheim.

The reversible electrodeposition allows for the modification of composition, dimensions, and form of plasmonic structures. As shown in **Figure** **3**a(i), the reversible full‐color plasmonic cells and displays achieved by electrochemically controlling the structure of a gold (Au)/silver (Ag) core‐shell nanodot array were successfully integrated into a mechanical chameleon that can automatically blend with colored backgrounds.^[^ ^96^ ^]^ It presents the extracted reflecting peak positions versus depositing/stripping time in Figure 3a(ii). These results suggest the realization of the reversible quasi‐full visible‐color plasmonic cell, which can switch between any two color states in a matter of seconds. It demonstrates a fast color‐changing system that enables real‐time color conversion by responding to the background detection. Figure 3a(iii) shows an operational schematic of the “fast display”, where all plasmonic cell units are operated simultaneously, and Figure 3a(iv) shows the procedure of discoloration. The method is relatively durable and can be used for more than 200 cycles, and the refreshing rates can reach up to 1 Hz. However, the complex fabrication method, weak mechanical durability, and lack of practical prototypes are also the current limitations of this design.

The gap between the nanoparticles is also a key factor affecting the resonant frequency. Dynamic tuning is achieved by using a flexible substrate made of polydimethylsiloxane (PDMS) with mechanical deformation. Figure 3b(i) shows the tunable structural colors based on tensile substrates.^[^ ^97^ ^]^ The measured reflective spectra with different stretched lengths are shown in Figure 3b(ii). The insets are the scanning electron microscopy (SEM) images with different stretched lengths. The calculated corresponding CIE chromaticity diagram of the reflective spectra is shown in Figure 3b(iii). It can be observed that deformation occurs with the external force on the PDMS substrate. As a result, the SPR wavelength will dynamically tune as the period of structures on PDMS changes concurrently. Meanwhile, the macroscopic measurements have produced a color that ranges from green to fuchsia through the use of interference photolithography and etching techniques. As a consequence, this wide range of colors can serve as a color comparison for the external force. However, this reconfiguration lacks durability and will lose elasticity after repeated stretching.

These techniques provide high saturation and versatility for applications such as adaptive displays and active camouflage by enabling quick and reversible color changes throughout the visible spectrum. However, there are issues with scalability, durability, and cost when precise nanoscale production methods like electrodeposition or mechanical stress applications are used. In order to increase stability and operational lives, the next research should concentrate on developing fabrication techniques for large‐scale, flexible devices. Wearable technology and real‐time color‐matching devices are only two examples of potential applications that might be further expanded by investigating hybrid systems that combine plasmonic structures with functional materials like phase‐change compounds or stretchable substrates.

In a metal‐insulator‐nanohole (MIN) plasmonic cavity, the refractive index and extinction of the insulator (i.e., electrochromic oxide) can be actively changed to modify the plasmonic resonant frequency of the metal‐nanohole array and, consequently, its wavelength‐selective light reflection and transmission properties.

**Figure 4:**
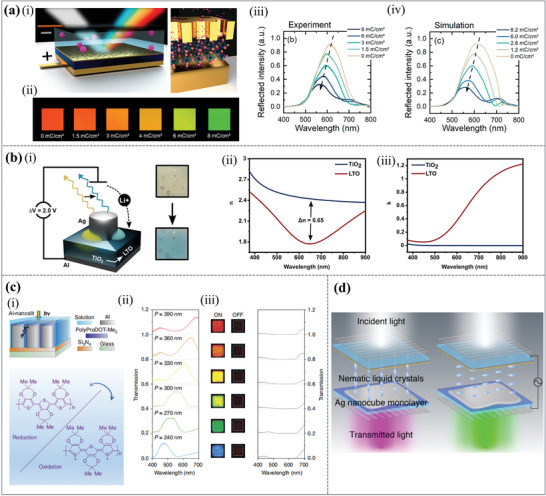
Dynamic structural colors are achieved by changing material properties of plasmonic structures. (a) Plasmochromic nanocavity dynamic light color switching. (i) Schematic of the plasmochromic device based on an electrochromic WO_3_ layer and a gold resonator (not to scale) and the cubic phase of WO_3_. (ii) Raw photos of the reflected colors at different stages of the lithiation process, each measuring 100 µm in lateral size. (iii) Experimentally obtained and (iv) the simulated reflectivity at various stages of the lithiation.^[^ ^98^ ^]^ Copyright 2020, American Chemical Society. (b) Dynamic structural colors driven by TiO_2_ electrochemistry. (i) Schematic diagram of the electrochemical lithiation process. (ii) Change in refractive index and (iii) absorption coefficient derived from electrochemical lithiation.^[^ ^99^ ^]^ Copyright 2022, American Chemical Society. (c) High‐contrast and fast electrochromic switching enabled by plasmonic structural colors. (i) Schematic diagram of a plasmonic electrochromic electrode incorporating an Al‐nanoslit array. Transmission spectra and micrographs for (ii) ON and (iii) OFF states of the polymer are displayed, respectively.^[^ ^100^ ^]^ Copyright 2016, Nature. (d) Schematic images of the proposed plasmonic color‐switching device.^[^ ^101^ ^]^ Copyright 2023, American Chemical Society.

Electrochromic oxides, such as tungsten oxide (WO_3_), provide active control over resonant conditions for plasmonic MIN‐based devices by allowing one to modify their refractive index (2.1−1.8) and extinction coefficient (0−0.5) upon the ion insertion. A plasmochromic color modulation device was made by using inorganic, electrochromic WO_3_ as the tunable dielectric in a MIN nanocavity, as shown in **Figure** **4**a(i),^[^ ^98^ ^]^ which was able to maintain 35% reflectance intensity and theoretically achieve a resonant wavelength modulation from 601 to 505 nm. Figure 4a(ii) are raw micrographs of the reflected color images taken with a CMOS camera. The images were captured at lithiation states ranging from 0 to 8 mC cm^−2^ throughout the lithiation process. In addition, the reflectance at each stage of lithiation obtained through experiments and simulations is shown in Figure 4a(iii) and (iv). The hue shift is accompanied by a similar drop in reflected intensity in both simulations and experiments. Consequently, for reversible wavelength shifts of more than 64 nm in the visible spectrum (552−616 nm), this adaptable plasmochromic device takes advantage of the sharp change in refractive index (2.1−1.8) and the corresponding extinction of electrochromic WO_3_ thin films. It also can predict color modulation of up to 100 nm, spanning from blue to red. However, though this method uses less power and switches quickly, it has a narrow tuning range.

Titanium dioxide (TiO_2_) is a highly versatile optical material, particularly suited for dynamic colors switching, owing to its ability to undergo reversible lithium‐ion insertion. This property is utilized in plasmonic nanocavities, where the electrochemical lithiation of TiO₂ into Li_0.5_TiO_2_ (LTO) induces a significant and tunable refractive index change.^[^ ^99^ ^]^ As illustrated in Figure 4b(i), this process requires an external voltage of only 2 V, making it highly energy‐efficient compared to many other dynamic optical systems. As shown in Figure 4b(ii),(iii), when anatase TiO_2_ is electrochemically lithiated to LTO, the absorption coefficient is less than 0.1 at blue wavelengths and the index change is 0.65 at 649 nm. Importantly, this refractive index modulation occurs with minimal optical absorption in the visible spectrum, preserving color vibrancy and optical efficiency. Its low absorption and high durability over repeated lithiation cycles further enhance its suitability for integration into advanced nanophotonic architectures.

Additionally, dynamic structural colors modulation can be achieved by directly altering the intrinsic material properties of nanostructures. This approach involves modifying parameters such as refractive index, extinction coefficient, or phase state through tuning methods, including electric fields, thermal changes, or chemical reactions.

Dynamic structural colors modulation is achieved by applying a small voltage, which alters the polymer's oxidation state, changing its absorption characteristics. As shown in Figure 4c, both monochromatic and full‐color fast switching with high optical contrast is demonstrated, based on the above concepts, using two ordinary electrochromic polymers, polyaniline (PANI) and poly (2,2‐dimethyl‐3,4 propylenedioxythiophene) (PolyProDOT‐Me_2_), integrated with periodic metallic nanoslit arrays.^[^ ^100^ ^]^ Figure 4c(i) displays the schematic diagram for the Al‐nanoslit electrode, and the oxidized and reduced chemical structures of PolyProDOT‐Me_2_. The experimentally observed optical transmission spectra of the manufactured Al‐nanoslit electrodes, along with the corresponding optical micrographs for both transmitting ON and absorbing OFF states of the polymer, are shown in Figure 4c(ii),(iii). We can clearly observe significant color changes between these two states. Furthermore, the plasmonic electrochromic electrodes demonstrated here exhibit switching speeds in tens of milliseconds, comparable to pixel switching speeds in commercial displays. This capability enables their use in complex applications requiring dynamic switching.

Combining a silver nanocube (AgNC) monolayer with a nematic‐phase liquid crystal (LC) layer results in electrical plasmonic color modulation, as demonstrated in Figure 4d.^[^ ^101^ ^]^ By integrating LC with negative dielectric anisotropy onto a densely packed AgNC monolayer, a color‐modulation LC/AgNC device was fabricated. When applying voltages ranging from 0 to 15 V, the LC molecules’ orientation changes, altering the average refractive index and dynamically tuning the plasmon resonance, so the transmitted color through the LC/AgNC device varied between green and magenta. The application of bias caused shifts in the transmission peak wavelengths due to the birefringence of LC. This spectral color shift resulted from the convolution of the transmission spectrum associated with the plasmon resonance of AgNC and the wavelength shift induced by the birefringence. Clear color modulation was achieved by leveraging the high plasmon resonance of AgNC. The facets of a crystalline AgNC are flat, and an integrated layer of closely packed AgNC produces a smooth surface for a plasmonic substrate, with their facets facing upward. This configuration of the AgNC monolayer suppresses alignment disorder, making it suitable for LC devices.

Fast response and high saturation are benefits of using electrochemical control of the material properties of nanostructures to produce SPR/LSPR dynamic structural colors. However, poor material stability and complicated manufacturing are drawbacks. Future developments in large‐area fabrication techniques, multifunctional regulating technology, and novel materials with low loss and high durability could increase the application potential of dynamic structural colors in the domains of display, sensors, and intelligent optics.

Structural color modulation can also be accomplished by altering the incident light conditions, such as the illumination light's polarization state, in addition to altering the material properties and structural parameters.

**Figure 5:**
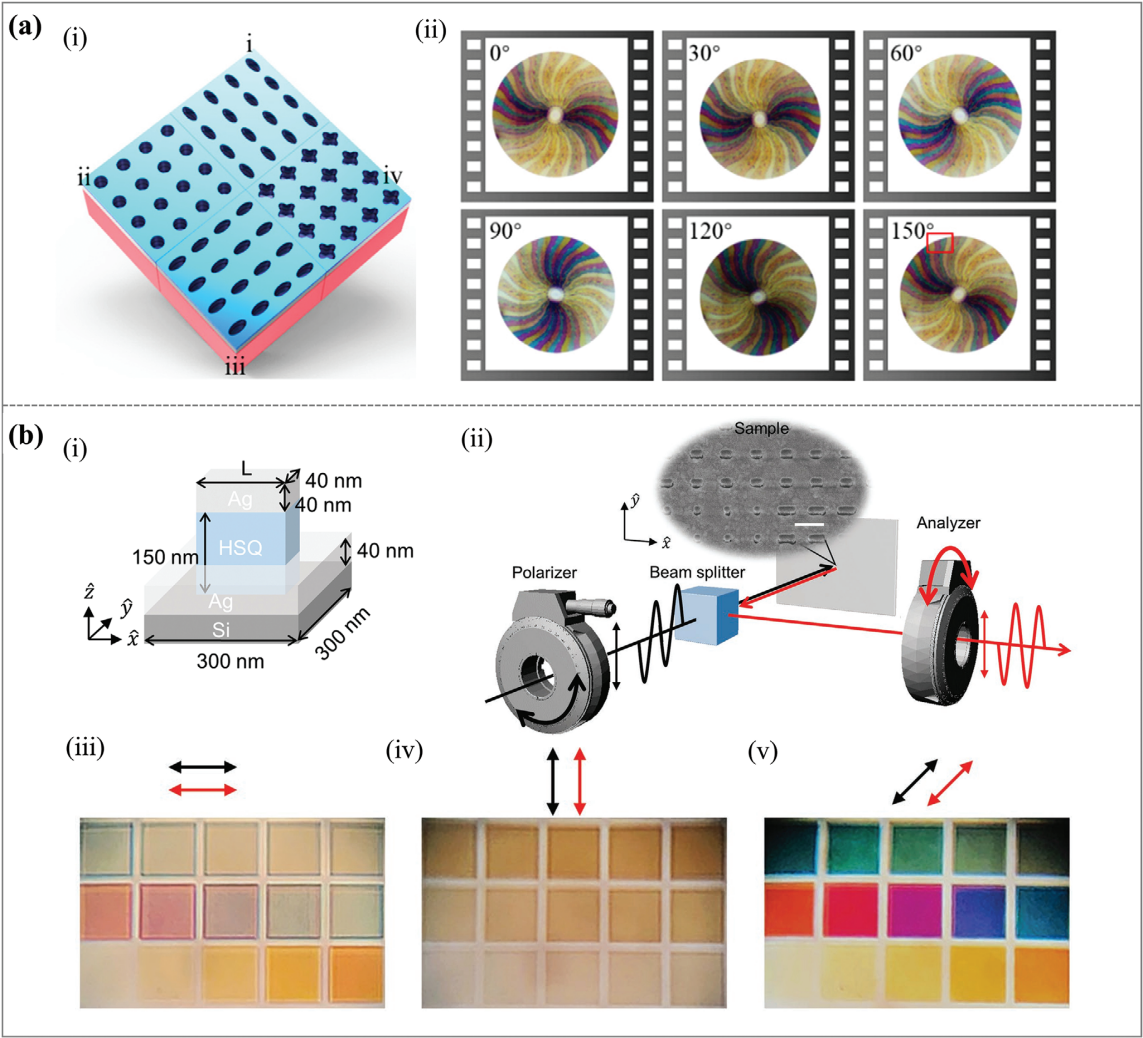
Dynamic structural colors are achieved by changing the incident light conditions. (a) Polarization‐sensitive subtractive structural color is used for information encoding and dynamic display. (i) The coding units that are employed to encode QR‐code patterns. (ii) Dynamic polarization‐resolved display for color printing patterns.^[^ ^102^ ^]^ Copyright 2020, Elsevier Ltd. (b) Polarization‐controlled dynamic structural color devices. (i) Schematic diagram of the structure of a single Ag nanorod. (ii) Readout schematic using two polarizers. The readout system is positioned (iii) horizontally, (iv) vertically, and (v) at 45° to the nanostructure, corresponding to the observed color images.^[^ ^103^ ^]^ Copyright 2023, Wiley‐VCH GmbH.

As shown in **Figure** **5**a(i), a polarization‐sensitive subtractive structural color based on elliptical Al/amorphous silicon (a‐Si)/Al nanostructures was designed, which revealed a wide color gamut covering the whole cyan, magenta, and yellow (CMY) color system.^[^ ^102^ ^]^ The elliptical structure, characterized by the shape anisotropy, facilitates the printing of colors with polarization sensitivity, resulting in different color hues from the same area through changes in incident light polarization. Furthermore, a dynamically controllable polarization‐dependent color disk was displayed using meticulously designed elliptical nanostructures. The polarimetric response of the colorful disk irradiated by the linearly polarized light varies from 0° to 150° in a 30° increment, as shown in Figure 5a(ii). The results demonstrate that the animation effect is produced by the colorful components rotating as the incidence polarizer changes continually.

Meanwhile, a scheme to achieve tunable structural colors using polarization‐sensitive plasmonic nanorods is proposed for encryption and color display applications. As seen in Figure 5b(i), the structure is made up of Ag nanorods that have been placed on dielectric pedestals above a reflective Ag mirror.^[^ ^103^ ^]^ The asymmetric form of the nanorods causes anisotropic reactions, and their periodic array pattern inhibits diffraction at visible wavelengths. Depending on the size of the nanorod and the polarization of the light, the geometry allows for exact control over color creation. The schematic diagram of the readout using two polarizers is shown in Figure 5b(ii). The two polarizers are parallel (Figure 5b(iii)), vertical (Figure 5b(iv)), and rotated by 45° (Figure 5b(v)) to the nanostructure to correspond to the observed color change. Consequently, this capacity is used to develop polarization‐controlled chromo‐encryption systems, in which polarizations function as keys for quaternary systems that encode and decode data. Its preparation is challenging nonetheless. Furthermore, its colors are mostly determined by structural geometry and polarization, which limits the possibility of obtaining some vivid hues without the use of other materials.

**Figure 6:**
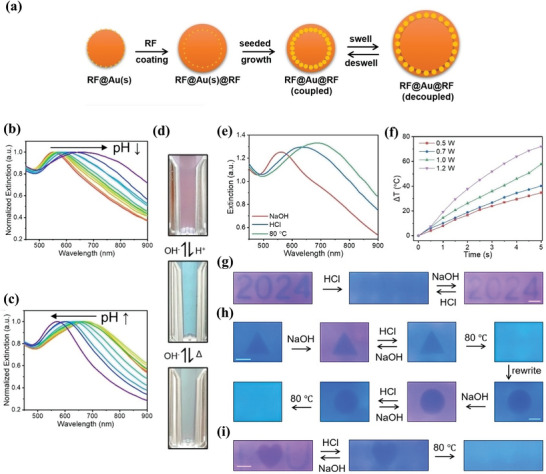
Dynamic structural colors are achieved by changing external environmental variables. (a) Schematic diagram of the synthesis process of AuNPs. Extinction spectra of a typical (b) NaOH‐treated and (c) HCl‐treated RF@Au@RF dispersion. (d) Digital images of color change in response to pH changes and thermal treatment. (e) RF@Au@RF/gel film extinction spectra after treatment with heat, HCl, and NaOH. (f) The curve picture of temperature changes over time with lasers of different powers. (g) Digital images of switchable patterns in digital form that display the digits “2024” produced on RF@Au@RF/gel film following 532‐nm laser illumination. (h) Digital pictures of rewriteable patterns that, in basic and acidic environments, display a variety of colors and can be removed by heating. (i) Digital pictures of the anti‐counterfeiting movie that display various data in various scenarios.^[^ ^104^ ^]^ Copyright 2024, Wiley‐VCH GmbH.

External environmental variables are still the key factors in achieving dynamic structural colors based on SPR/LSPR resonances. Resorcinol‐formaldehyde (RF) polymer layers respond to pH and temperature changes, driving reversible swelling and deswelling. The use of gold nanoparticles (AuNPs) confined within these swellable polymer colloidal spheres can achieve dynamic structural color modulation through plasmonic coupling.^[^ ^104^ ^]^ The schematic diagram of the synthesis process of AuNPs is shown in **Figure** **6**a. Figure 6b,c show the spectral changes corresponding to the reversible response of AuNPs to pH changes. Meanwhile, the reversible color change diagrams under NaOH, HCl, and thermal treatments are shown in Figure 6d. This result demonstrated that RF polymer layers, which react to pH and temperature changes by causing reversible swelling and deswelling, may be used to control AuNPs plasmon coupling to produce color change. Moreover, when added to polymer hydrogel films, the RF@Au@RF hybrid nanostructures show reversible color changes in response to external stimuli, including heat, HCl, and NaOH, simulating the behavior seen in colloidal dispersions (Figure 6e). Under 532‐nm laser irradiation, as illustrated in Figure 6f, the embedded AuNPs produce localized heat via photothermal conversion, which causes the RF matrix to condense and permits quick, region‐selective plasmonic coupling. Ultimately, the hybrid film's photothermal action and responsive plasma coupling were combined to create dynamic anti‐counterfeiting patterns (Figure 6g–i).

The benefits of this approach include great reversibility, good cyclic stability, multimodal stimulation regulation, and quick response speed. However, this method still needs to be optimized in terms of durability and dynamic structural color regulation range due to the material properties of the polymer matrix and AuNPs; at the same time, its potential for large‐scale application is limited by its sensitivity to environmental conditions and complicated preparation process.

Dynamic structural colors based on SPR/LSPR resonances have the advantages of high sensitivity, a wide color gamut, and multifunctional regulation. By adjusting the size, shape, and gap of nanostructures, the refractive index of the substrate medium layer, material properties, the incident light conditions, and the external environmental variables can accurately control the resonant wavelength to achieve high resolution and high saturation dynamic structural colors. There are various ways to regulate it, including electric fields, mechanical deformation, polarization, pH, and temperature, which are suitable for display, anti‐counterfeiting, sensors, and biomedicine. However, such devices also face limitations such as high material cost, optical loss issues, mechanical and thermal stability, large‐scale fabrication complexity, and scaling challenges. Future development directions include the development of low‐loss and high‐stability nanomaterials, optimizing structural design to achieve a wider range of dynamic regulation, and combining multimodal response technologies (such as light, electricity, and heat) to improve the performance and adaptability of the device, thereby expanding its application potential in smart displays and optical communication.

### 3.2 FP‐Based Structural Colors

According to Section 2.2, changing the thickness and material properties of the structural layers will affect the resonant frequency of the FP cavity and thus the achieved colors. It is therefore possible to design dynamic structural colors by influencing the resonance using tuning methods (e.g., femtosecond laser‐processed, temperature, electric fields, lithiation, micro‐electro‐mechanical system (MEMS), thermal oxidation, hydrogenation, etc.).

**Figure 7:**
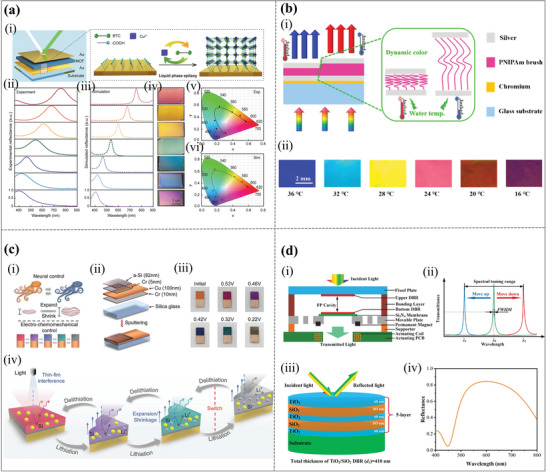
Regulating spectrum via the cavity length adjustment in dielectric layers. (a) Monolithic MOF‐based MIM resonator construction with adjustable bandwidth at visible frequencies. (i) Schematic diagram showing the deposition of SURMOFs to build a MIM resonator; the light source is typically incident upon a planar surface with any polarization. (ii) Experimental and (iii) simulated reflection spectra of the MOF‐based MIM resonator. (iv) Optical pictures, obtained by activating the MIM resonator at normal incidence, with reflected colors that match the spectra in Figure 7(a)(ii). The dashed/solid lines with arrows in the CIE 1931 chromaticity map correspond to the corresponding coordinate distribution of the (v) experimental and (vi) simulated reflection, and they show the evolution trend of resonators's bandwidth.^[^ ^105^ ^]^ Copyright 2023, American Chemical Society. (b) Dynamically tunable color display based on MIM resonator with polymer brush insulator layer. (i) Dynamic structural color‐changing mechanism of the MIM FP resonance cavity. (ii) Corresponding dynamic photographs taken by an optical microscope.^[^ ^106^ ^]^ Copyright 2019, American Chemical Society. (c) Bioinspired controllable electro‐chemomechanical coloration films. (i) The color shift in cephalopods serves as an inspiration for the reversible coloration process under electro‐chemomechanical control, which neutrally regulates the volume of cells or proteins. (ii) Diagram showing the changeable coloring film's layered structure. (iii) Images taken during the Li^±^ intercalation process of tunable coloring films at various voltages. (iv) Mechanisms of the chromatic and achromic process.^[^ ^107^ ^]^ Copyright 2018, WILEY‐VCH Verlag GmbH & Co. KGaA, Weinheim. (d) Widely and linearly tunable, electromagnetically actuated MEMS FP filtering chips. The basic structure is shown in (i), where the moveable plate can be pushed to move up or down by electromagnetic force by varying the current in the actuating coil. This results in a modulation of the FP cavity length and (ii) a corresponding change in the transmitted wavelength. (iii) Schematic diagram, and (iv) its simulated reflectance over the whole visible band.^[^ ^108^ ^]^ Copyright 2023, Optical Society of America.

One important factor influencing the realization of structural colors in FP resonances is thickness, which primarily dictates the resonance wavelength and interference circumstances. It is possible to generate structural color shifts in the entire spectrum by carefully varying the thicknesses of dielectric layers. For example, a monolithic metal‐organic‐framework (MOF)‐based metal‐insulator‐metal (MIM) resonator with tunable bandwidth was reported to boost both dynamic optical filtering and active chemical sensing by laser‐processing microwell arrays on the top metal layer.^[^ ^105^ ^]^ The design of the surface‐mounted metal‐organic‐framework (SURMOF)‐based MIM resonator, which has a high brightness in the visible spectrum and broad color gamut tuning, is shown in **Figure** **7**a(i). By altering the number of spraying cycles for the SURMOFs growth, Figure 7a(ii) displays the adjustable reflection of the Au/SURMOFs/Au resonator with a major reflection band at *λ* = 408, 474, 543, 587, 644, and 690 nm. The MOF‐based MIM resonator exhibited a greater reflectivity of over 95% compared to the MOF‐based photonic crystals that have been described. The reflection spectra for the planar MIM resonator with various insulator thicknesses, as determined by a finite‐difference time‐domain (FDTD) simulation, are shown in Figure 7a(iii). Typically, a plane wave strikes the upper surface of the MIM resonator, causing a broad light source to be excited by the resonator's incidence and revealing vivid hues ranging from blue to red. The findings of the experiment and the simulated reflection corresponded quite well. Additionally, optical pictures of the MOF‐based MIM resonator revealed a changeable hue from blue to red (Figure 7a(iv)). The constructive interference caused angle‐dependent reflection in the MIM resonator that were reported. A higher‐index dielectric spacer may be able to lessen the angle dependence of the colors since the refraction angle in a high‐index film stayed small over a wide range of incident angles. The CIE 1931 color space (Figure 7a(v)) showed that the MOF‐based MIM resonator obtained a wide range of colors and outstanding color purity, which was consistent with the simulated results (Figure 7a(vi)). The significant step forward in integrating optical filtering and chemical sensing with advanced MOF‐based resonator designs. The next could focus on simplifying fabrication, broadening analyte ranges, and enhancing environmental stability.

Instead of chemically swelling materials, thermo‐optic tunable materials integrated with nanostructures can also achieve dynamic structural colors. As shown in Figure 7b(i), a simple MIM trilayer FP resonance cavity with a poly (N‐isopropylacrylamide) (PNIPAm) brush layer as a responsive element was reported as a thermal‐induced colorimetric response platform.^[^ ^106^ ^]^ Unlike other photonic crystals or plasmonic nanostructures, its straightforward construction eliminates the need for complex manufacturing techniques. As seen in Figure 7b(ii), the PNIPAm brush layer swells quickly in response to changes in the external temperature, causing the FP resonance cavity to instantly change color visually. Thus, structural colors could be dynamically adjusted with a rapid response rate and good reproducibility by varying the external temperature. Considering the diverse nature of stimulus‐responsive polymer brushes, this design strategy presents a general approach to achieving a wide range of functionalities. Furthermore, it introduces a novel perspective for the advancement of dynamic structural color‐changing materials. This FP resonance cavity will provide new opportunities in application fields such as optical sensors, display devices, and activity monitors.

In addition to the single‐factor regulation, various forms of regulation that work together could also have a brilliant color‐changing performance. A bioinspired coloration film using a nanoscale a‐Si layer deposited on a reflective metal substrate was designed.^[^ ^107^ ^]^ Tunable chromogenic qualities throughout a wide visible spectrum are made possible by these coloring films, which precisely manipulate reversible lithiation and delithiation behaviors. Simultaneous changes in chemical components and film thickness during electrochemical processes significantly alter conditions for destructive interference. Figure 7c(i) shows the reversible color display of the interference coloration film under electro‐chemomechanical control. The structure of the pristine interference coloration film is shown in Figure 7c(ii). By sputtering the film onto a silica substrate, the intercalation and deintercalation processes of lithium ions (Li^+^) successfully stabilize the film. Besides the orange‐red color of the initially controllable coloration film, Figure 7c(iii) shows five representative colors—magenta, medium purple, dark blue, dark cyan, and gray (achromic)—that are readily apparent throughout the Li^±^ intercalation process at different voltages. The electro‐chemomechanical mechanism is presented in Figure 7c(iv). Thus, the coloration mechanism caused by the thickness and intrinsic qualities is confirmed by the appropriate model based on electro‐chemomechanical coupling effects (refraction index and optical absorptivity).

Our group proposed a large‐aperture, widely and linearly tunable, electromagnetically actuated MEMS FP filtering chip (MEMS‐FPFC) for visible and longwave infrared spectral imaging (LWIRSI).^[^ ^108^ ^]^ To achieve the design and construction of the suggested MEMS‐FPFC, a multi‐field coupling simulation model was constructed, and the wafer‐scale bulk micromachining method was used. Figure 7d(i) gives the fundamental structure, by changing the current in the actuating coil, the movable plate can be driven by the electromagnetic force to move up or down, which leads to the modulation of FP cavity length, and the transmitted wavelength is thus changed correspondingly in Figure 7d(ii). As shown in Figure 7d(iii), the 5‐layer TiO_2_/silica (SiO_2_) distributed Bragg reflectors (DBR) (*n*_h_:*n*_l_ = 2.2:1.46@600 nm) were used to balance the transmittance and full‐width at half‐maximum (FWHM) of the MEMS‐FPFC. Simulated reflectance is shown in Figure 7d(iv), and the total thickness of DBRs (*d*_1_) is computed to be 410 nm. Moreover, we expected that the tensile stress of TiO_2_ and the compressive stress of SiO_2_ could counteract to some extent to keep the film stress of the DBR stack in an acceptable range.

The benefits of using modulated cavity thickness to modify the FP resonance frequency in order to generate dynamic structural colors are low power consumption, a wide color gamut, high precision, high saturation, and reversibility. This technique works well in domains including intelligent optical filtering, dynamic display, and optical sensors. Its limited response speed (often seconds), material durability (e.g., fatigue due to electrochemical expansion), and restricted regulatory range (material and structural constraints on thickness variations) still limit its performance. Furthermore, the color's brightness and repeatability may be diminished by optical loss and sensitivity to the outside world. In order to enhance performance and expand application scenarios, future optimization prospects include the creation of composite cavities, multimodal regulation, and high‐durability materials.

Simultaneously, material properties are key factors in determining FP resonances and thus the resulting colors. Leveraging tuning methods, such as electric fields and temperature changes, to dynamically alter material properties offers a versatile approach to achieving dynamic structural color tuning. For example, phase‐change materials, electrochromic polymers, or thermochromic coatings can be integrated into FP structures to enable reversible and precise modulation of the optical response, expanding their functionality for applications in displays, sensors, and smart coatings.

**Figure 8:**
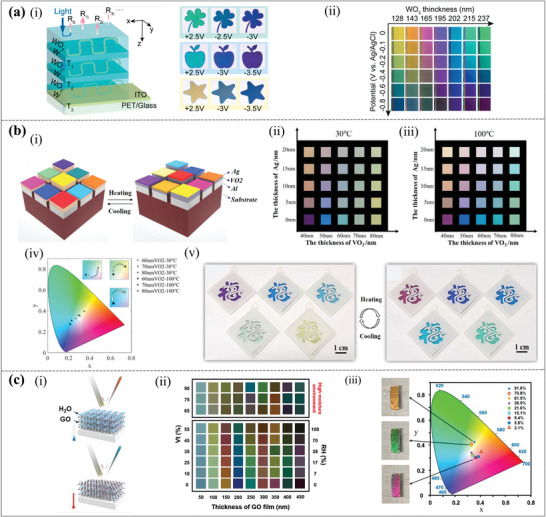
Dynamic structural colors based on inorganic electrochromic materials. (a) Electrochromic metamaterials of metal‐dielectric stacks for multicolor displays. (i) Left: schematic of light propagation in periodic W‐WO_3_ MMDM‐structured EC electrodes at normal incidence; Right: optical images of the MMDM‐based EC device captured at different color states. (ii) Color variations in MMDM film according to different WO_3_ voltages and thicknesses.^[^ ^109^ ^]^ Copyright 2021, American Chemical Society. (b) Dynamic structural colors using the phase‐change material VO_2_ and an ultrathin asymmetric FP cavity. (i) A schematic diagram and illustration of the suggested dynamic structural colors for theoretical study. With the bottom Al layer at 50 nm, a color palette of the reflecting hues as functions of the thicknesses of the Ag and VO_2_ layers at (ii) 30 °C and (iii) 100 °C was recorded. The size of each sample is 6 mm * 6 mm. (iv) CIE color coordinates for the samples at various temperatures and VO_2_ thicknesses. (v) The color patterns used for decoration before and after heating.^[^ ^110^ ^]^ Copyright 2021, Optical Society of America. (c) Tunable TGMs with iridescent color. (i) Schematic for the color‐tuning principle of TGMs. (ii) TGM color palettes that were artificially created using varying GO film thicknesses and water amounts. (iii) Coordinate experiment findings in the CIE 1931 color space.^[^ ^111^ ^]^ Copyright 2022, American Chemical Society.

Inorganic electrochromic (EC) materials, like WO_3_, which is extensively utilized, can conveniently modify their optical properties via electrochemical processes. **Figure** **8**a(i) demonstrated multilayered metal‐dielectric metamaterials (MMDMs) as a new family of inorganic‐based EC materials to achieve dynamic alternation among multiple colors with high contrast and high color purity.^[^ ^109^ ^]^ By greatly increasing the confinement of the incident light at specific optical frequencies, this was achieved structurally. The richer color modification capacity of the MMDM electrodes was due to the ion implantation into the top WO_3_ layer. The three‐stack W‐WO_3_ MMDM electrode, consisting of W and WO_3_, exhibited a similar trend of reflection peaks. Under various applied potentials, it showed a more varied color modulation from pale green to medium aquamarine, light sea green, turquoise, slate blue, and dark orchid. As shown in Figure 8a(ii), the color change is a function of WO_3_ thickness and applied voltage to achieve a full‐color palette covering the entire visible range. The colors exhibited by MMDM electrodes shift from yellow to green as the WO_3_ film thickness increases from 128 to 237 nm (a clockwise change in the CIE color coordinate chart). Regardless of WO_3_ thickness, the MMDM electrode progressively modulates its color along the direction of the hypsochromic shift as the applied negative voltage rises from 0 to −0.8 V. This enables the realization of an extensive color palette. The proposed structure signifies a burgeoning field of EC metamaterials characterized by exceptional color saturation, potentially paving the way for the next generation of reflective EC devices.

Phase‐change materials also offer facile adjustment of optical constants and can serve as dielectric layers in FP cavities. An asymmetric ultrathin FP‐type structure with a VO_2_ cavity our team proposed to realize vivid subtractive flexible structural colors with stable color‐switching performance, good flexibility, and low sensitivity to the angle of incidence, as shown in Figure 8b(i).^[^ ^110^ ^]^ Because of its reversible monoclinic‐rutile phase transition, which enables improved color modulation above or below the transition temperature, the VO_2_ phase‐change material layer is essential and adds to the color‐changing performance. The color hue, saturation, and brightness can be altered by simply changing the thicknesses of VO_2_ and top Ag layers, as shown in Figure 8b(ii). The color palette after heating to 100 °C is shown in Figure 8b(iii), and the CIE 1931 color space (Figure 8b(iv)) provides a clear illustration of the color‐changing performance before and after heating. In other words, by varying the thicknesses of the top Ag and VO_2_ layers, the color hue, saturation, and brightness can be altered. Additionally, the above color performance may be changed both before and after the heating. Furthermore, normal thin‐film deposition techniques can be used to quickly produce the above switchable structural colors on rigid or flexible substrates. Thereby, this allows a large‐scale, low‐cost implementation on practical devices. Here, our team created structural color samples on Si substrates with varying VO_2_ thicknesses, which were then placed in the auspicious cloud molds. By adjusting the heating temperature, the color‐switching patterns were achieved, as shown in Figure 8b(v). Instead of using VO_2_ phase‐change material, we can also use thermos‐optic tunable materials integrated with plasmonic nanostructures to realize the dynamic structural colors. This approach balances simplicity, flexibility, and functionality. Future work could explore expanding stimuli responsiveness (e.g., electrical or optical triggers) and improving durability for broader applications.

Recently, the concept of dynamically tunable thermochromic graphene metamaterials (TGMs) was proposed and demonstrated.^[^ ^111^ ^]^ These materials can achieve continuous color tunability within the range of 380–800 nm with fast response times of less than 100 ms. The TGMs are made of a flexible metal substrate covered in an ultrathin layer of graphene oxide (GO). The water content of the GO film can be dynamically changed by external thermal energy, enabling TGM color modulation. A conceptual representation of the operating principle of TGMs with varying water contents inside the GO film is shown in Figure 8c(i). As shown in Figure 8c(ii), under varying humidity circumstances, it exhibits the simulated color palettes of TGMs with varying GO film thicknesses in the range of 50–450 nm. The color coordinates in the CIE 1931 color space of TGMs with a 355 nm GO film at various humidity levels at 20 °C are shown in Figure 8c(iii). When RH% varies from 3% to 91% at 20 °C, the colors of TGMs with the 355 nm GO film can span a significant portion of the CIE XYZ color space. This result clearly demonstrates that TGMs exhibit significant color changes from purple to green, and eventually to yellow, depending on humidity levels. In summary, TGMs are cost‐effective, scalable, and flexible, allowing for versatile integration with curved surfaces.

Combined with the above, high precision, a wide color gamut, and reversibility are benefits of using modulating material qualities (such as refractive index, extinction coefficient, or phase change) to alter the FP resonance frequency in order to generate dynamic structural colors. However, phase change materials may have stability issues under long‐term cycles; the control range is constrained by the amplitude of the change of material properties; it is challenging to cover the entire visible spectrum; and the response time is typically dictated by the rate of change of material properties, which may be slow. Furthermore, this approach typically depends on a single tuning method (such as an electric field or temperature), which restricts the development of multifunctional applications. Its application potential in display, sensing, and intelligent optical devices can be increased in the future by introducing multifunctional composite materials and optimizing material structure, which will also increase control efficiency and stability.

**Figure 9:**
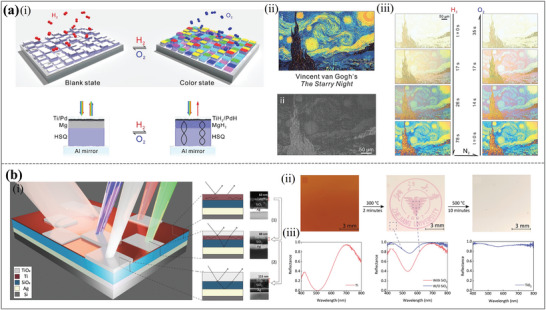
Dynamic structural colors through chemical properties. (a) Dynamic structural colors based on the chemical phase change via hydrogenation and dehydrogenation using FP resonators. (i) Diagram of the stepwise FP resonator‐based dynamic color display. (ii) A computer‐generated layout of Vincent van Gogh's The Starry Night based on the overview SEM image of the fake display and the color database retrieved from the palette. (iii) A dynamic display of the artwork that alternates between color and black/white printing.^[^ ^112^ ^]^ Copyright 2017, American Chemical Society. (b) Large‐scale, panchromatic structural color manipulation via thermal trimming. (i) The schematic for the cavity. (ii) The demonstration of information‐revealing and destroying processes. (iii) The reflectance spectra corresponding to (ii).^[^ ^113^ ^]^ Copyright 2021, Wiley‐VCH GmbH.

Furthermore, dynamic regulation of the spectrum can also be achieved by changing properties of the top material of the FP cavity, such as by chemical reaction. **Figure** **9**a(i) shows a novel dynamic structural color printing scheme using magnesium‐based pixelated FP cavities.^[^ ^112^ ^]^ Magnesium (Mg) exhibits unique metal and dielectric transitions with regulated hydrogenation and dehydrogenation, allowing for different blank and color states from the pixelated FP resonators. Following such a scheme, they initially displayed dynamic Ishihara plates, in which only hydrogen serves as an information‐decoding key to read out the encrypted images. In this case, Mg serves as both a surrounding substance and a component of dynamic pixel construction. Vibrant color shifts from individual FP resonators are made possible by the Mg layer's reversible transition between a reflecting metal state and a dielectric hydride state upon hydrogen absorption and desorption. The computer‐generated layout of Vincent van Gogh's The Starry Night, as seen in Figure 9a(ii), was created using the palette's color database and the constructed display's overview SEM image. Additionally, Figure 9a(iii) shows some transformations between black/white printing and color printing. This scheme can achieve high contrast and a wide color gamut, and this work lays a solid foundation for advancing dynamic optical technology and realizing high‐performance displays using innovative material transformations and FP resonances.

A simple and practical strategy supporting wafer‐scale and panchromatic structural color post‐trimming is shown in Figure 9b(i).^[^ ^113^ ^]^ The suggested gadget may be precisely adjusted from red to blue, spanning the entire visible spectrum, by only managing a thermal oxidation process. It was accomplished by regulating the oxidation degree of the top Ti layer based on a MIM cavity. Wafer‐scale structural color post‐trimming was possible with this method, and the trimming range could cover the whole visible region. A unique “burn to read” and “burn after reading” structural color device was made possible by the slow and precisely regulated transition from the Salisbury screen cavity to the Gires–Tournois cavity during this heating phase, as well as the low loss of TiO_2_ in the top layer. The demonstration of the information‐revealing and destroying process is shown in Figure 9b(ii). Figure 9b(iii) shows the corresponding spectra for each period. It is appropriate for consumer‐level anti‐counterfeiting because of this fascinating property. Additionally, it offers a straightforward method for structural color creation and trimming at the wafer size. However, material limitations and color stability issues also restrict its large‐scale practical application.

High precision and a wide color gamut are benefits of the technique of producing dynamic structural colors by altering the material properties in response to chemical processes (such as thermal oxidation or hydrogenation) of the top material. However, the limited variety of materials that can be used and the fact that this type of approach depends on chemical reactions occurring within or on the surface of the material limit its adaptability. Simultaneously, the chemical reaction process typically has a slow response speed, making it challenging to meet the demands of high‐speed dynamic modulation; environmental factors like temperature and humidity can have an impact on color stability. Repeated reactions can also result in mechanical fatigue or material deterioration, which can impair long‐term performance. Optimizing material stability and reaction efficiency is a crucial area for future research and development for this kind of dynamic structural color design.

In summary, the benefits of dynamic structural colors based on FP resonance design include low power consumption, a wide color gamut, high precision, high saturation, and reversibility. They are appropriate for large‐scale and commercial fabrication and can handle dynamic adjustment requirements from ultraviolet to near‐infrared. However, its performance limitations include slow response speed, insufficient material cycle stability, limited control range, and sensitivity to environmental conditions (such as humidity and temperature). In order to increase the application potential in display, sensors, anti‐counterfeiting, and intelligent optical devices, future development directions include creating new materials with high durability, achieving multimodal control and high‐speed response, optimizing flexible and large‐area fabrication technologies, and enhancing environmental stability and device intelligent design.

### 3.3 Mie‐Based Structural Colors

According to Section 2.3, by varying the structural geometric parameters, material properties, surrounding medium, incident light conditions, the structural colors produced by Mie resonances can be made dynamic. Based on Mie resonances, these parameter settings offer dynamic structural color flexibility and variety.

**Figure 10:**
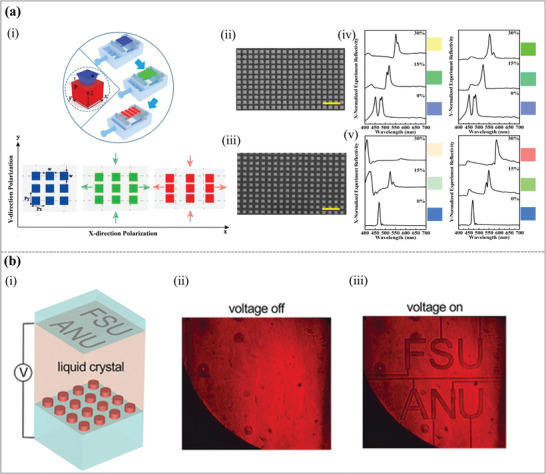
Dynamic structural colors by varying the structural geometric parameters and material properties. (a) A stretchable all‐dielectric metasurface. (i) The stretchy TiO_2_ metasurface schematic image. The TiO_2_ metasurface's top‐view SEM images have (ii) *p* = 300 nm, *w* = 230 nm, and (iii) *p* = 320 nm, *w* = 210 nm. The scale bar is 1 µm. (iv) The strain‐dependent reflection spectra. (v) How external strain affects reflection spectra. Illustrations are in corresponding colors.^[^ ^114^ ^]^ Copyright 2019, American Chemical Society. (b) A dielectric metasurface enables electrically tunable transparent displays for visible light. (i) Schematic diagram of the structure of the electrically controlled LCs metasurface cell. Real‐color images of the metasurface at (ii) voltage off, and (iii) voltage on were recorded in the transmission.^[^ ^115^ ^]^ Copyright 2019, American Chemical Society.

The nanostructure with PDMS as the substrate can adjust the geometric parameters (lattice period) by mechanical stretching. As shown in **Figure** **10**a(i), a TiO_2_ metasurface designed based on this scheme to achieve dynamic structural colors.^[^ ^114^ ^]^ And the system simultaneously adjusts the resonant wavelengths for both polarizations, ensuring polarization‐insensitive color modulation. The top views of the SEM images of the prepared two different TiO_2_ metasurfaces with geometric parameters are shown in Figure 10a(ii) *p* = 300 nm, *w* = 230 nm, and (iii) *p* = 320 nm, *w* = 210 nm. Figure 10a(iv),(v) show the measured reflectance spectra of the two structures under x‐polarization and y‐polarization as the strain increases, as well as the corresponding color changes. As the stress increases, the color of both structures changes significantly and can cover the entire visible spectrum while being insensitive to polarization. Therefore, this work establishes a robust platform for stretchable, polarization‐insensitive metasurfaces with broad applications in emerging photonics technologies. Although this work provides a solid foundation for stretchable, polarization‐insensitive metasurfaces, the color change is restricted to strain‐induced adjustments, which may not provide as fine control as other external stimuli, and the performance is dependent on uniform and precise stretching, which may restrict the scalability of large‐area devices.

At the same time, altering the material properties to provide dynamic structural colors is another interesting method. As shown in Figure 10b(i), an electrically tunable transparent display based on a dielectric metasurface is proposed, which can achieve dynamic structural colors in the visible light range.^[^ ^115^ ^]^ By adding nematic LCs to the dielectric metasurface, the effective refractive index of the metasurface can be controlled by using the anisotropic characteristics of the LCs molecules to alter their orientation when exposed to an external electric field. By shifting the metasurface's resonant frequency in response to this change in refractive index, the color of reflected or transmitted light can be dynamically controlled. Specifically, applying a voltage can cause the resonance peak to shift by more than twice its line width, demonstrating significant tuning capabilities, as shown in Figure 10b(ii),(iii). In general, this paper shows that electrically tunable transparent displays based on dielectric metasurfaces can dynamically control color in the visible light spectrum. This has a wide range of potential applications, but response time and manufacturing process still require improvement.

**Figure 11:**
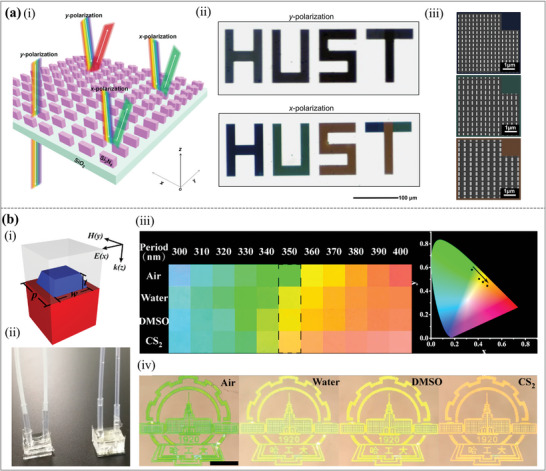
Dynamic structural colors by varying the incident light conditions and surrounding medium. (a) Switchable structural colors via polarization on all‐dielectric metasurfaces. (i) Schematic diagram of all‐dielectric metasurfaces. (ii) The “HUST” bright‐field optical images. (iii) The matching SEM images of the panel's metasurfaces made out of nanorods.^[^ ^116^ ^]^ Copyright 2023, American Chemical Society. (b) Real‐time tunable colors from microfluidic reconfigurable all‐dielectric metasurfaces. (i) Schematic picture of one unit of the TiO_2_ metasurface. (ii) TiO_2_ metasurfaces integrated with PDMS microfluidic chips. (iii) Left: TiO_2_ metasurfaces’ experimentally recorded reflection colors in various solvents observed under a bright‐field microscope; Right: CIE coordinates of the matching hues in the dashed box. (iv) Color images of the Harbin Institute of Technology logo that change dynamically.^[^ ^117^ ^]^ Copyright 2018, American Chemical Society.

Variations in external excitation conditions (incident light) will also be a key factor in achieving dynamic structural colors. A polarization‐switchable structural color technology based on an all‐dielectric metasurface was proposed, the structural diagram is shown in **Figure** **11**a(i).^[^ ^116^ ^]^ Dynamic switching of structural hues can be accomplished by creating polarization‐sensitive all‐dielectric nanostructures (such as nanorods and nanocrosses) and exploiting their selective reaction to incident light in various polarization states, as shown in Figure 11a(ii). The matching SEM images are shown in Figure 11a(iii). High resolution, high saturation dynamic structural colors, multi‐mode display, and concealment capabilities are all possible with this metasurface, which is also appropriate for anti‐counterfeiting and information storage. On the other hand, its production complexity is considerable, the background reflection issue may reduce the encryption effect in some modes, and the viewing angle sensitivity may impact the display consistency.

Furthermore, the structural colors can be tuned with the change of the surrounding environment. For instance, a simple method of integrating the TiO_2_ metasurfaces into the microfluidic channel was proposed to realize real‐time tunable structural colors.^[^ ^117^ ^]^ The distinct structural colors can be rapidly tuned by injecting different solvents. As shown in Figure 11b(i), the system consists of a TiO_2_ nanoblock array with a lattice constant p on an ITO glass substrate. Figure 11b(ii) shows TiO_2_ metasurfaces integrated with PDMS microfluidic chips. And Figure 11b(iii) shows the bright‐field microscope images corresponding to the periodic changes of the metasurface experimentally measured in different solvents. After research, it was found that the device will dynamically adjust the color pair as the refractive index of the surrounding environment changes. The logo of Harbin Institute of Technology with dynamic structural colors changes designed using this structure is shown in Figure 11b(iv). In addition to having a large color gamut and excellent saturation, the system suggested in this work offers great resolution, strong reversibility, and a quick response time (16 ms). This approach's viability for large‐scale fabrication is constrained by its reliance on intricate procedures like electron beam lithography. Its reliance on particular solvents may compromise the practical applications capacity to adapt to changing environmental conditions. Develop low‐cost, scalable fabrication processes, optimize material and structural design to achieve a wider range of dynamic control capabilities, and explore other dynamic control mechanisms to replace microfluidic liquids, such as gas or solid‐state response, to further expand its application potential.

Mie resonances can be controlled to produce dynamic structural color regulation with high saturation and high purity. It has low loss, a wide color gamut, high resolution, and versatility, making it appropriate for a variety of applications, including optical sensing, dynamic display, and anti‐counterfeiting encryption. Its response is fluid and reversible, and its regulation techniques range from altering the surrounding medium to altering the structural size or the material properties. However, current devices have several limitations, such as environmental sensitivity, angle sensitivity, fabrication complexity (like electron beam lithography (EBL)), and inadequate scalability in large‐area and flexible applications. Future developments should focus on developing low‐cost, scalable fabrication techniques, investigating new dynamic response materials (like phase change materials or responsive polymers), improving the device's environmental stability and response speed, and combining multimodal regulation technology to increase its potential applications in complex optical regulation and smart displays.

### 3.4 Others

In addition to the aforementioned dynamic structural colors based on SPR, LSPR, FP, and Mie resonances, other controlling methods can be realized by Bragg scattering, leaky mode resonances, and Fano resonances or integrating multiple structural principles.^[^ ^94^, ^118^, ^119^ ^]^

**Figure 12:**
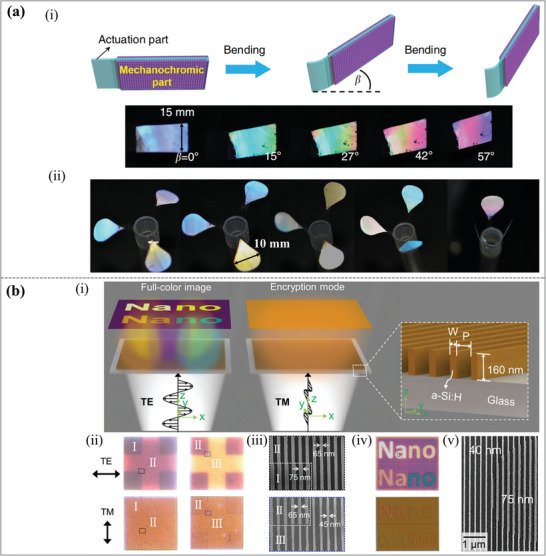
Dynamic tunability of structural colors based on Bragg scattering and leaky mode resonances. (a) An actuator powered by molecular channels produced dynamic structural colors. (i) Schematic illustration and photograph of a stimuli‐responsive color‐changing actuator. (ii) A smart flower thus fabricated, which can change its shape and color depending on the humidity.^[^ ^120^ ^]^ Copyright 2018, Nature. (b) Leaky mode resonances‐based polarization encryption that makes use of a‐Si:H NGs. (i) Schematic illustration of the proposed color pixel. (ii) Bright‐field microscopy images under TE and TM polarized illumination. (iii) SEM images of (ii). (iv) Brightfield microscopy images of the “NanoNano” under TE and TM polarized illumination. (v) SEM images of (iv).^[^ ^121^ ^]^ Copyright 2020, De Gruyter.

An ambient‐driven actuator was designed that utilized the inherent nanoscale molecular channels within a commercial perfluorosulfonic acid ionomer (PFSA) film.^[^ ^120^ ^]^ The actuator was fabricated through the simple solution processing and achieved rapid response, self‐adaptiveness, and exceptional stability. In response to the vapor stimuli, PFSA films were patterned on a soft, inert substrate (polyethylene terephthalate film) to create a variety of shapes, such as a 2D roll or a 3D helical structure. The surface was chemically modified to create a kirigami‐inspired single‐layer actuator that could manage personal humidity and heat through macroscale geometrically designed features. This led to the development of a bilayer stimuli‐responsive actuator with the multicolor switching capability. The film experienced the bending actuation in relation to the exposed, developing PFSA segment, as seen in **Figure** **12**a(i). A PFSA film was partially Ag‐plated on both sides to create this flexible multilayer composite film. The plated area was then coated with SiO_2_ nanoparticles that were well‐ordered and contained in PDMS. When the relative humidity varies, it displays a spectrum of deformations, expressed in degrees. The composite, multilayer film portion (covered by SiO_2_ nanoparticles/PDMS and Ag plating) changed color at a constant viewing angle as the degree of bending increased or decreased with respect to neutral. As demonstrated in Figure 12a(ii), where color was determined by the external humidity, the PFSA‐based system was utilized to exhibit smart color‐changing flowers due to its huge actuation amplitude, fast response time, and relatively wide range of attainable colors. The control mode is simple, and will not destroy the microstructure organization, and has relatively high durability but also a limited range of color change.

In particular, Figure 12b(i) illustrates a method for producing transmissive polarization‐encrypted structural color by utilizing hydrogenated amorphous silicon (a‐Si:H) nanogratings (NGs) based on leaky mode resonances.^[^ ^121^ ^]^ When the polarization state of the incident light switches from TE (perpendicular to the grating) to TM (parallel to the grating), the color changes from full color to a uniform orange. Figure 12b(ii) shows the images under TE and TM polarization. The microscopic color photographs clearly display bright magenta and yellow cross patterns on dazzling purple and magenta backgrounds, respectively, under the illumination of TE‐polarized light. All three areas display the same encrypted orange hue when the incident light polarization changes to TM polarization. SEM images of the regions indicated by the rectangular boxes with dots in black and blue in Figure 12b(ii) are shown in Figure 12b(iii). In addition, Figure 12b(iv) designs a “NanoNano” pattern encryption application that can achieve dynamic switching under polarization. Its SEM image is shown in Figure 12b(v). This mode conversion makes the structural color highly dependent on polarized light, thus realizing encryption function.

In summary, these two papers explore the technologies of achieving dynamic structural colors through molecular channel driving and a‐Si:H NGs, respectively. The molecular‐channel‐driven actuator achieves reversible mechanical deformation through humidity or solvent stimulation. Its advantages are low power consumption, strong environmental responsiveness, and suitability for flexible devices, but it is highly dependent on the environment and has limited long‐term stability. Polarization‐encrypted, high‐resolution full‐color images use polarization state switching to achieve full‐color encryption. It has the advantages of high resolution (25400 dpi), a wide viewing angle, and easy manufacturing, but is limited by the spectral range and high dependence on polarization. Future application directions of both include flexible display, anti‐counterfeiting technology, dynamic camouflage, information encryption, and smart surface decoration.

## 4 Outlook and Conclusion

**Table** **2** provides a comprehensive breakdown of the various modulation techniques for dynamic structural colors discussed in this analysis, enabling both a general understanding and a detailed comparison of their advantages, limitations, and applications. While significant progress has been made in this field, challenges such as large‐area fabrication, practical applications, and performance optimization remain. Nonetheless, structural color technologies hold immense promise to transform industries such as color printing, intelligent displays, and imaging filters in the near future. By drawing on recent advancements, this review offers a forward‐looking perspective on the opportunities and challenges for dynamic structural colors, paving the way for their broader adoption in next‐generation applications.

**Table 2:** Comparison of dynamic structural colors based on different resonance modes.

Resonances	Key factors	Tuning methods	Advantages	Limitations	Applications	Refs.
SPR/LSPR	1) Geometric parameters 2) Material properties 3) Incident light conditions 4) External environment	1) Electric fields 2) Mechanical deformation 3) Polarization 4) pH 5) Temperature	1) Fast response 2) High saturation 3) High sensitivity 4) Nano‐level precision 5) Versatility	1) High material cost 2) Fabrication complexity 3) Optical loss issues 4) Mechanical and thermal stability 5) Scaling challenges	1) Camouflage 2) Anti‐counterfeiting 3) Tunable filter 4) Biomedical imaging 5) Sensing 6) Smart building	[96, 97, 98, 99, 100, 101, 102, 103, 104]
FP	1) Thickness 2) Material properties	1) Femtosecond laser‐processed 2) Temperature 3) Electric fields 4) Lithiation 5) MEMS 6) Hydrogenation 7) Thermal oxidation	1) Low power consumption 2) Wide color gamut 3) High precision 4) High saturation 5) Reversibility	1) Material durability 2) Low response speed 3) Environmental sensitivity	1) Smart windows 2) Dynamic displays 3) Spectral filters 4) Adaptive camouflage 5) Biomedical imaging 6) Decorative materials 7) Sensors 8) Wearable technology	[105, 106, 107, 108, 109, 110, 111, 112, 113]
Mie	1) Geometric parameters 2) Material properties 3) Surrounding medium 4) Incident light conditions	1) Mechanical stretching 2) Electric fields 3) Polarization 4) Different solvents	1) High saturation 2) High purity 3) Low optical loss 4) Wide color gamut 5) High resolution 6) Versatility	1) Environment sensitivity 2) Fabrication complexity 3) Angle dependence 4) Inadequate scalability	1) Sensors 2) Dynamic display 3) Anti‐counterfeiting 4) Encryption	[114, 115, 116, 117]
Bragg scattering	Monolayer membrane bending and deformation	Humidity	1) Versatility 2) Fast response 3) Bio‐inspired design 4) Material availability 5) Multi‐dimensional deformation capability	1) Environmental dependence 2) Low color range 3) Low load capacity	1) Flexible sensors 2) Stealth technology 3) Decorative materials	[120]
Leaky mode resonances	Incident light conditions	Polarization	1) Fast switching 2) High resolution	1) Low spectral range 2) High manufacturing precision requirements	1) Encryption 2) Anti‐counterfeiting 3) High‐resolution multi‐functional display 4) Security tag	[121]

### 4.1 Prospect and Developments

#### 4.1.1 Fabrication Goes Cheaper in a Larger Area

Presently, structural colors were mainly fabricated by high‐cost approaches such as EBL, focused ion beam milling (FIB),^[^ ^122^ ^]^ etc., which all hindered the practical application of structural colors seriously.^[^ ^123^ ^]^ For instance, the practical size of exhibited color printing does not surpass 1 mm^2^ because of the incredibly slow writing speed of EBL. Thus, it is necessary to study the approaches for mass production to meet the practical needs. The researchers have proposed many different potential methods,^[^ ^124^, ^125^, ^126^, ^127^, ^128^ ^]^ such as inkjet printing,^[^ ^129^ ^]^ roll‐to‐roll nanoimprint,^[^ ^130^, ^131^ ^]^ nanotransfer printing with nanoimprint‐based planarization,^[^ ^132^ ^]^ resonant laser printing,^[^ ^133^, ^134^ ^]^ and so on.^[^ ^135^ ^]^

**Figure 13:**
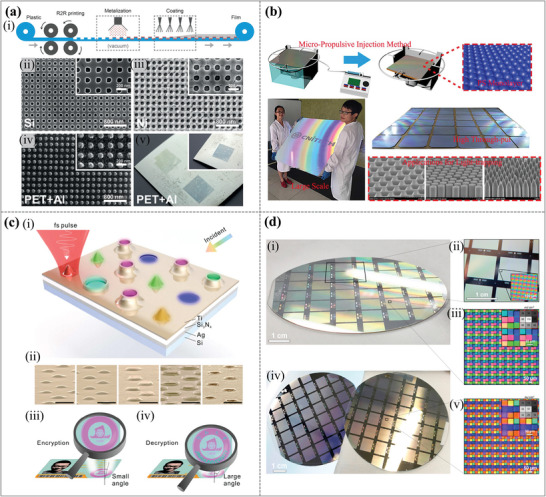
The large‐scale production method of structural colors. (a) Roll‐to‐roll nanoprinting. (i) A diagram showing the expected roll‐to‐roll procedure. (ii) Si master SEM pictures. (iii) Nickel master SEM pictures. (iv) SEM pictures of aluminum‐topped roll‐to‐roll polymer replication. (v) Images of the completed, uncoated A‐PET polymer surface.^[^ ^131^ ^]^ Copyright 2016, WILEY‐VCH. (b) Initial stage for the injection of polystyrene colloids over the water surface.^[^ ^137^ ^]^ Copyright 2015, American Chemical Society. (c) On‐chip steganography in angular anisotropy by femtosecond (fs) pulse laser‐printed nanovolcanoes. (i) Diagrammatic representation of printed nanovolcanoes illuminated by oblique lighting, using a fs pulse and scattered color. (ii) SEM images of nanovolcanoes showing how their morphology changes over time. On‐chip picture steganography encryption and decryption at small and large incident angles are shown schematically in (iii) and (iv), respectively.^[^ ^133^ ^]^ Copyright 2018, American Chemical Society. (d) Wafer‐scale grayscale‐to‐color multispectral filter array fabrication. (i) A macro lens‐taken, zoomed‐in image of a 3‐inch wafer with ≈32 9‐band MSFAs (using second‐order resonances) (ii). (iii) A transmission optical micrograph of an alternative wafer region. (iv) An optical micrograph of one MSFA and (v) a photograph of two 3‐inch MSFA wafers (using first‐ and second‐order resonances).^[^ ^138^ ^]^ Copyright 2019, American Chemical Society.

Roll‐to‐roll printing is an emerging technology in the field of manufacturing micro‐ and nanoscale patterning that has attracted interest due to its inherent advantages of low cost, high throughput, and large‐area patterning.^[^ ^131^ ^]^ It offers scalable mass production of long‐lasting structural colors in addition to the coating's protective qualities. Schematic of the anticipated roll‐to‐roll process is shown in Figure 13a(i). Additionally, SEM images of Si master, nickel master, and roll‐to‐roll polymer replication with Al on the top are shown in Figure 13a(ii)‐(iv), respectively. Figure 13a(v) is an image of the completed A‐PET polymer surface without coating for 0.5 × 0.5 mm (blue) and 300 nm period (green) areas. To create color metasurfaces of industrial utility, they fused plasmonic metasurfaces based on LSPR modes with polymer replication. A high‐speed roll‐to‐roll nanoimprint lithography (R2RNIL) on flexible plastic substrates was proposed, demonstrating a true R2RNIL process in which polymer patterns down to 70 nm feature size were continuously imprinted on a flexible web.^[^ ^136^ ^]^ Because it also uses a mechanical embossing technique, the R2RNIL technology inherits the high‐resolution feature of standard NIL; nevertheless, the speed of nanopatterning has risen by at least one or two orders of magnitude. Large‐area patterning of nanostructures is the main goal of this approach, which tackles a problem that hinders nanopatterning in many real‐world applications.

As seen in Figure 13, a high‐throughput surface texturing procedure for optical and optoelectronic devices was described, based on a large‐area self‐assembly of nanospheres using an inexpensive micropropulsive injection (MPI) method.^[^ ^137^ ^]^ The MPI approach enables the direct formation of hexagonally structured nanosphere arrays (NAs) into a well‐organized monolayer on the water surface, subsequently transferred to prepared substrates. Capable of producing 3000 wafers per hour, this technique is ideal for large‐scale solar production, offering flexibility in creating periodic nanotextures on device surfaces. Leveraging laser writing's flexibility, various straight micropillars with diverse cross‐sections can be easily fabricated. Under the pulsed laser irradiation, strong on‐resonance energy absorption rapidly elevates lattice temperatures to over 1200 K within 1 ns, forming the basis for resonant laser printing. This enables surface energy‐driven morphological changes and color adjustments in real‐time.

As shown in Figure 13c(i), femtosecond‐laser‐printed nanovolcanoes exploiting subwavelength and 3D morphologies were proposed to allow the experimental demonstration of on‐chip steganography from angular anisotropy.^[^ ^133^ ^]^ By employing pulsed laser contact with MIM structures, this technique precisely regulates transient local heating and subsequent mass redistribution of molten metal layers. As shown in Figure 13c(ii), precise control of laser parameters in the focal profiles and pulse fluences enables the printing of nanovolcanoes with varying morphologies and heights correlating to different resonance frequencies or scattering colors. Consequently, embedded encrypted information that is only visible at the predetermined decoding angle may be present in a school badge composed of matching nanovolcanoes (Figure 13c(iii)‐(iv)). As a result, a laser printing method of this kind might be employed for the large‐scale, scalable manufacturing of imprints measuring millimeters or more.

A manufacturing process that enables cost‐effective wafer‐level fabrication of customized multispectral filter arrays (MSFAs) based on grayscale (analog) lithography using a single lithographic processing step is presented in Figure 13d.^[^ ^138^ ^]^ Through the user‐controlled, dose‐modulated exposure scheme, they generated spatially variant 3D MIM cavities covering the entire pixel area using the grayscale lithography. This was demonstrated using grayscale‐mask‐based photolithography (G‐PL) to showcase scalability for practical volume applications, alongside grayscale electron beam lithography (G‐EBL) as a proof of concept. Using a streamlined single lithographic processing step (grayscale‐to‐color) to fabricate extremely efficient, narrowband, highly customizable MSFAs with full pixel coverage. This approach is scalable to wafer‐level fabrication for real‐world usage. Using the mask‐based PL grayscale‐to‐color technique, ≈32 MSFAs with nine spectral bands (3 × 3) were patterned on a 3‐inch wafer (Figure 13d(i)). A zoomed‐in region captured with a macro lens and optical micrograph (transmission mode) of different regions on the wafer are shown in Figure 13d(ii),(iii), respectively. Selected from batches of multiple wafers, this illustrated wafer demonstrates the versatility of the bespoke MSFA approach with narrower second‐order FP‐type resonances (i.e., thicker final resist thickness); we can incorporate first‐ and second‐order modes with ease by varying the flood exposure dose, as more other wafers (Figure 13d(iv),(v)) demonstrate.

The above methods solve the large‐area manufacturing to a certain extent, but they still have significant limitations. For the nanoimprinting method, the fabrication of the stamping mold is cumbersome and expensive. Laser writing is limited by the type of material; it can only be used for metal dielectrics or polymers, and some are limited to flexible or rigid substrates. Thus, there is still room to explore new methods for cost‐effective, large‐scale mass industrial preparation to meet the practical applications of dynamically tunable structural colors.

#### 4.1.2 Applications Go Broader in Functionality

At present, dynamic structural colors have shown wide application potential in display technology, anti‐counterfeiting, adaptive camouflage, encryption, sensors, smart windows, wearable devices, and biomedical imaging.^[^ ^93^, ^139^, ^140^, ^141^, ^142^, ^143^, ^144^, ^145^, ^146^ ^]^ Dynamic structural color regulation achieved through multiple stimuli such as light, electricity, heat, and mechanical stress has been used in high‐resolution dynamic displays, reversible encryption tags, and environmentally responsive decorative materials.

**Figure 14:**
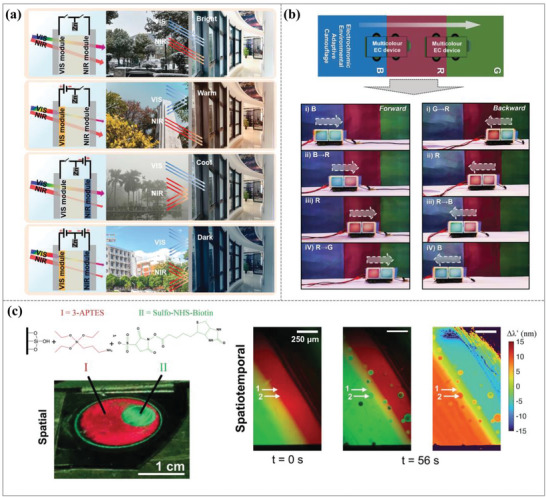
Various practical applications of dynamic structural colors. (a) Schematic schematic demonstrating the performance and workings of a multi‐color, four‐mode DESW in different modes.^[^ ^147^ ^]^ Copyright 2024, Wiley‐VCH GmbH. (b) A model car's schematic (top) and photos (bottom) show how the skin color changes in response to the different hues of the surroundings.^[^ ^148^ ^]^ Copyright 2024, Wiley‐VCH GmbH. (c) Dynamically resolved structural color sensing in both space and time.^[^ ^149^ ^]^ Copyright 2024, Wiley‐VCH GmbH.

For example, a multicolor, four‐mode, dual‐band, electrochromic smart window (DESW) has been designed, which can achieve a multifunctional integrated design of optical and thermal regulation by independently regulating the transmittance of visible light (VIS) and near‐infrared light (NIR). The DESW features four modes that can be utilized to modify the color based on weather conditions, as seen in **Figure** **14a**.^[^ ^147^ ^]^ Using two electrochromic modules is the basic idea: 1) Prussian blue analogues (MoOHCF) thin film module: the color superposition effect changes from yellow to black by altering the redox state, which also controls the VIS transmittance. 2) WO_x_ thin film module: modifying NIR transmittance by means of polaron electrochromic processes and plasma. Accurate dynamic modulation of VIS and NIR light is achieved by integrating these modules with the asymmetric zinc anode, which guarantees independent functioning and avoids voltage conflicts. Despite its many benefits, such as its unique multi‐color dynamic regulation, exceptional energy‐saving capabilities, robust cycle stability, and integrated energy recovery function, the DESW is appropriate for energy management and comfort enhancement in green buildings. The system can yet be improved in terms of response speed, energy recovery efficiency, and color aesthetics, though the intricate multilayer design might make large‐scale manufacture more difficult. Future research should concentrate on refining material qualities to increase durability and color range, streamlining production procedures to cut costs, and enhancing energy recovery efficiency to encourage broad use in sustainable energy and smart building applications.

Another excellent application for dynamic structural colors is adaptive camouflage. As shown in Figure 14b, an adaptive camouflage system designed by a new electrochromic polymer (PPBE) with a donor gradient molecular structure can respond to background color changes and has full color adjustability.^[^ ^148^ ^]^ The system exhibits outstanding cycling stability (≥5000 times), great coloring efficiency (395.1 cm^2^ C⁻¹), and quick switching (≈1.4 s). Its novel donor gradient design produces colorful switching at low voltage while improving molecular polarity and charge separation efficiency. Full‐tone coverage, real‐time reaction, and biomimetic adaptive capabilities are among the benefits; nevertheless, there are still drawbacks, including intricate molecular design, challenges with large‐scale production, and the requirement for energy efficiency improvement. In the future, it can be enhanced to accomplish wide‐ranging applications and broaden into domains like environmental sensors, dynamic textiles, and smart displays, all the while boosting energy efficiency to encourage the use of sustainable portable devices.

Additionally, Figure 14c shows a spatially and spatiotemporally resolved dynamic structural color sensor.^[^ ^149^ ^]^ The dual‐band supercolor dynamic structural colors approach of mesoporous Si metamaterials, which controls the refractive index distribution of the mesoporous Si rugate filter to produce high‐sensitivity red‐to‐green color conversion, is the basis for this application. This technique offers a novel framework for high‐resolution, real‐time sensing without spectral scanning by utilizing dual laser irradiation and quantitative color mapping. High responsiveness, precise quantitative mapping, multi‐modal measurement capabilities, and affordable, real‐time spatial and temporal sensing applications are some of its benefits. However, this approach has several limits when it comes to expanding the color spectrum of applications, optimizing complex optical systems, and choosing materials. Future directions for research include developing portable integrated sensing modules, increasing research on the long‐term environmental stability of mesoporous Si to support its widespread use in chemical, biological, and environmental monitoring fields, improving selectivity through surface chemical functionalization, and broadening the color gamut range and application scenarios.

#### 4.1.3 Performance Goes More Practical and Beyond

The resolution of plasmonic structural colors generated by Au, Ag, and other precious metal materials can be reached at 100000 dpi. However, this approach is costly due to the use of noble materials, with the Ag being prone to oxidation. Furthermore, the color gamut of certain additive or subtractive colors is limited, failing to encompass the entire visible spectrum. Moreover, the performance of FWHM, the polarization state, and incident angle invariance also need to be improved. For the dynamic structural colors, the modulation by LC can realize fast adjustment, but its thickness is larger, which cannot be bent and lacks flexibility. Mechanical deformation can achieve a wide modulation range, but repeated use significantly reduces its fatigue life. Color‐changing mechanisms like electrochemistry or chemical reactions necessitate complex environments and exhibit slower refreshing rates. The thermochromic system based on vanadium dioxide is limited to changing from one color to another, offering limited color‐changing capabilities. Thus, there are still some challenges for the present dynamic structural colors. It is essential to enhance comprehensive performance in the following areas: 1) Regarding color and spectral aspects, the goal is to achieve vibrant structural colors with a wider gamut, narrower FWHM, larger viewing angles, and higher spatial resolution. 2) For dynamic tunability, new active control modulations are needed to achieve diverse colors across a broader wavelength range, with superior spectral selectivity, distinct switching capability, stable cycle performance, high color contrast, and rapid refreshing rates. 3) In other aspects, achieving structural colors with high flexibility, low energy consumption, long lifetimes, and cost‐effectiveness is conceivable. This approach can advance structural colors toward practical applications, going beyond traditional properties and driving technical innovations in various fields.

### 4.2 Prospect and Development

In conclusion, structural colors exhibit numerous superior properties not achievable by pigment colors, including high color quality, a wide color gamut, high spatial resolution, compatibility with CMOS technology, and environmentally friendly. This review outlines the fundamental concept, classification, and physical realization of structural colors, highlights several notable examples, and speculates on potential applications and future development trends. The mechanisms of color generation, including SPR, LSPR, FP, and Mie resonances, are discussed. This review then introduces the use of different tuning methods to change the key factors (geometric parameters, material properties, incident light conditions, external environment, etc.) in generating color in different resonance modes to achieve dynamic structural colors. It further evaluates the performance, advantages, and drawbacks of various technologies, focusing on both the potential and limitations of diverse dynamic structural colors. Lastly, the review presents an outlook and suggestions for large‐area fabrication strategies, practical applications, and performance enhancements for dynamic structural colors. Ultimately, despite the significant progress made in the field, it is recognized that this is merely the inception of its accelerated development. With the cumulative research efforts, structural colors will permeate various aspects of our lives in the near future.

## Conflict of Interest

The authors declare no conflict of interest.

## Author Contributions

All authors have accepted responsibility for the entire content of this manuscript and approved its submission.
